# Mimicking early life-forms in the lab

**DOI:** 10.7554/eLife.108470

**Published:** 2025-08-13

**Authors:** Maarten Lubbers, Dennis Claessen

**Affiliations:** 1 Microbial Sciences, Institute of Biology, Leiden University Leiden Netherlands

**Keywords:** protocells, microfossils, archaean eon, origin of life, biofilms, microfossil morphology, Other

## Abstract

Studying the growth of bacteria without cell walls in an artificial environment can shed new light on the proliferation of primitive life-forms billions of years ago.

**Related research article** Kanaparthi D, Westall F, Lampe M, Zhu B, Boesen T, Scheu B, Klingl A, Schwille P, Lueders T. 2025. On the nature of the earliest known lifeforms. *eLife* **13**:RP98637. doi: 10.7554/eLife.98637.

What did life look like billions of years ago? How did the earliest known life-forms proliferate? To answer these questions – which continue to intrigue scientists across many disciplines – we must travel back roughly 3.5 billion years. Although precious little direct evidence has survived since then, some ancient rock formations have retained traces that may reveal how early life-forms emerged and evolved. Two key geological formations offer valuable clues: the Pilbara Greenstone Belt in Australia and the Barberton Greenstone Belt in South Africa. These ancient rocks have remained largely unchanged for billions of years, preserving structures interpreted as fossilized cells, or microfossils, with carbon compositions that are consistent with the carbon coming from a biological source (Walsh, 1992; Schopf et al., 2018).

It is thought that early life-forms likely lacked many of the structural features found in modern prokaryotic cells, such as the cell wall, so direct comparisons with present-day prokaryotes are difficult and potentially misleading (Szostak, 2017). However, one way to study how primitive cells might have behaved is to use wall-deficient bacteria as experimental models. These bacteria, which grow and proliferate without a cell wall, resemble the presumed architecture of early life-forms and provide a tractable system to explore non-canonical modes of proliferation (Errington et al., 2016). Cell division in these bacteria depends on simple biophysical processes, such as increased membrane synthesis, rather than the more complicated process of binary fission that is employed by most prokaryotes (Mercier et al., 2012; Svetina, 2009). This makes wall-deficient bacteria an attractive platform for investigating how early cells may have grown and potentially left behind fossil-like traces. Now, in *eLife*, Dheeraj Kanaparthi, Tillmann Lueders and colleagues report how they have engineered a bacterium called *Exiguobacterium* strain-Molly into a wall-deficient state to mimic the proliferation of primitive life-forms billions of years ago (Kanaparthi et al., 2025).

To recreate the conditions in which early life may have thrived, Kanaparthi et al. carefully simulated an ancient environment. Since most microfossils would have thrived in coastal marine settings with surface temperatures estimated to be in the range 26–35 °C (Knauth, 2005), the wall-deficient bacteria were cultured at 30 °C in a medium enriched with Dead Sea salt. In these conditions, the bacteria grew to become much larger than their original size. Furthermore, they proliferated via two processes – by forming internal daughter cells, or by forming a string of external cells (Figure 1). Intriguingly, the structures formed by the bacteria as they proliferated closely resembled the shapes of microfossils found in ancient rocks.

**Figure 1.**
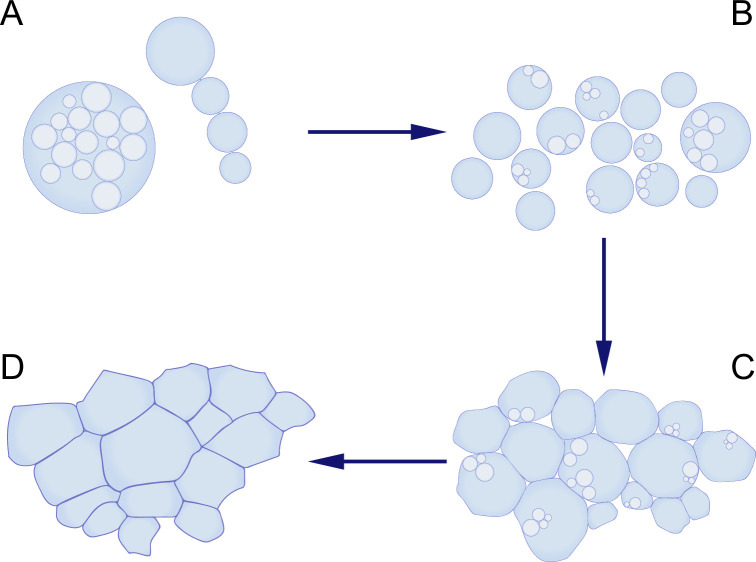
Using bacteria without cell walls to mimic the proliferation of early life-forms. (**A, B**) Kanaparthi et al. engineered a bacterium called *Exiguobacterium* strain-Molly (EM) into a wall-deficient state called EM-P, and found that these cells proliferated by forming internal daughter cells (**A**, left), or by forming a string of external cells (**A**, right). The cells then formed biofilms (**C**), which subsequently transformed into honeycomb-shaped mats (**D**), which resemble some of the structures seen in microfossils.

Image credit: Maarten Lubbers.

Fossils typically form when an organism dies and is rapidly buried by sediment. These sediments then harden into rock, where minerals replace the original organic material – a process that can take millions of years. To investigate how a primitive cell might degrade and become encrusted in salt, Kanaparthi et al. conducted experiments that ran for 28 months.

First, the researchers found that the bacterial cells formed multiple layers of cells as they proliferated, eventually forming a biofilm. Then, as the membranes of the cells broke down during lysis, the debris accumulated and began to form layered fabric-like structures. These layers were eventually expelled from the biofilm, and expanded to create continuous sheets that enclosed large populations of cells, resembling honeycomb-shaped mats.

Over time, Kanaparthi et al. observed the biofilms transitioning into a solid crust, which they suggest forms by the gradual adsorption of positively charged ions onto the negatively charged surfaces of the biofilm. Given that coastal marine environments often contain higher salt concentrations due to evaporation, the researchers propose that a similar encrustation process may have contributed to the preservation of early life-forms in the fossil record.

Based on their findings, Kanaparthi et al. – who are based at the Max Planck Institute for Biochemistry, the University of Bayreuth, Ludwig Maximilian University and other institutes in Germany, France, China and Denmark – suggest that the cells giving rise to early microfossils may have lacked a cell wall, resembling the engineered bacterial cells observed in their study. While these primitive cells likely lacked machinery for replicating, or for controlling their shape, Kanaparthi et al. argue that they may have developed basic mechanisms for energy conservation. Notably, the researchers found that bacterial morphologies varied depending on environmental conditions, which led them to suggest that the shapes of ancient microfossils were primarily shaped by external environmental factors rather than by genetic regulation.

Overall the this work provides a new experimental perspective on how early life-forms may have proliferated and been preserved, and offers valuable insights into the earliest stages of cellular evolution.
